# Extracts from the Introductory Lecture Delivered at the Opening of the Session of the Medical College of Georgia, on the First Tuesday in November, 1865

**Published:** 1866-07

**Authors:** Thomas S. Powell

**Affiliations:** Professor of Obstetrics and Diseases of Women and Children


### ARTICLE III.

*Extracts from the Introductory Lecture delivered at the opening of the session of the Medical College of Georgia, on the first Tuesday in November*, 1865, *by* THOMAS S. POWELL, M. D., *Professor of Obstetrics and Diseases of Women and Children.*

This is no ordinary occasion. It is an era in the life of the Atlanta Medical College. For four years her walls have been filled with sick and wounded soldiers. Erected as a retreat where the theories of medicine could be quietly taught, it has been used for the practical purposes of the profession. Her halls, once vocal with the busy hum of student life—the earnest lecture, the gay jest, the merry laugh, have echoed the groan of the wounded soldier dying away from home, from kindred and from family.

Our Faculty have been separated during the period referred to. Some have been engaged ministering to the wants of a suffering army—their places of labor transferred from the lecture-room to the hospital; others have been engaged in the less conspicuous but still arduous duties of private practice.

All, we trust, have done their duty in the different fields in which they have labored, and through the merciful and kind care of an overruling Providence, the lives of all have been spared.

All, save two, are still here, with renewed energy, and with lofty purpose, determined to do their duty, to the best of their ability, in fitting you for the responsible duties of your profession.

It is with profound regret I have to announce that two of our number have been compelled, on account of impaired health, to resign their chairs. It is with sadness and disappointment that we part with them. We had hoped that the growing fame of the one, and the sage wisdom and experience of the other, would long reflect honor on our institution. The comparative youth and brilliant career of the late Professor of Anatomy, as a teacher and a surgeon, led us to expect for him a long life of honorable usefulness among us, and a steadily increasing fame and reputation.

It is the earnest hope of his former colleagues, that his health, shattered by the exposure of the battlefield and his close application to hospital duty, may soon be restored, and that he will speedily return to the profession for which he is so eminently fitted. If, in the providence of God, this should be the case, we predict for him a brilliant and successful future. In regard to our honored friend, the late Professor of the Science and Practice of

Medicine, I would say that it is still our hope that his voice may occasionally be heard within the walls of the Institution to which he has been devoted, and around which his affections still cling, as we still claim him as one of us, if he is not able to be with us, having had assigned him by the Faculty the honorary position of Emeritus Professor.

In the darkness which it has pleased an all-wise Providence to bring upon him, he has our deepest sympathy, and we earnestly hope that he may be richly endowed with the light of that Spirit which will comfort him here, and illuminate his soul's pathway to a brighter world on high. May he be resigned to the sad dispensation which has befallen him, in the hope of a blessed immortality.

With respect to the gentlemen who have been selected to occupy the vacant positions, I should, perhaps, be lacking in delicacy and good taste, were I to say much in their favor. I would, nevertheless, remark that they are in every particular worthy to occupy the chairs which have been so ably filled, and by the faithful performance of the duties of their respective positions, they will no doubt prove themselves capable and worthy successors to honored predecessors.

This is an important era to you, gentlemen. It is especially important in the case of those of you who have never as yet attended a medical course. You have recently chosen your profession in life. It is a noble and philanthropic calling—one, as you know, requiring many sacrifices. Practiced with a proper appreciation of its duties, it is refining and exalting in its tendencies, and wins the respect and confidence of all intelligent communities. All the human sympathies are excited; there are constant opportunities for doing good; frequent calls for self-denial. You, looking forward with the imagination of youth, perhaps, see only the flowers in your pathway—bright suns, roseate hues or unclouded skies; verdant hills and luxuriant fields; friends to assist, and admirers to applaud—eagerly grasping, in anticipation, the laurel wreath of future distinction. But who can foretell your destiny? Far be it from me, however, to utter one word that would repress, in the slightest degree, the ardor of aspiring youth. But I feel that I would not properly discharge my duty as an instructor, as a friend to the country at large, if I did not warn you against commencing the study of a profession so highly responsible as the one in which you are now about to be engaged, without a full determination to use all your exertions to take advantage of all the opportunities presented for medical instruction; if I did not tell you that you had chosen this day a course of life full of thorns, hardships, sacrifices and difficulties; if I did not tell you that a toilsome pathway lies before you; and if I did not tell you at the same time, that at every step of the ascent you will find cheering compensation for the laborious effort incessantly necessary.

Many of you, owing to the stern life you have led, the cares, the dangers and vicissitudes of soldiers' duties, are better prepared for the realities of life that await you, than young gentlemen of your age usually are. You well know that in the life of a soldier the early training is all-important. Those men who have had the severe drill and careful training of a well conducted camp of instruction, are much better prepared for the active campaign than those who have no such training. It is all-important that you be well drilled here—it is all-important that you begin properly; your future success depends on your devotion to your studies here. It is necessary that you begin with correct principles and rules of action.

It is almost certain that the plan of study, the mode of thought, the peculiar habits of investigation and observation adopted by you here, will be continued in after life. If you observe and think closely now, you will observe and reason closely hereafter. The student must not skip his difficulties; he must conquer them. He must begin by times this noble habit, this only guarantee of ultimate success, while the opportunities are in his power.

Once contract a careless method of study, an eagerness to get on without having heaved the lead every inch of progress; without having, step by step, felt his way; without digesting each lecture, and assimilating it to the store of knowledge already thus laid up; once let a man's ostensible improvement outstrip his intrinsic and real one; let his knowledge, so far as it reaches, be unstable and only half acquired, and the man is undone. Each fresh conquest and acquisition must be secured forever to the mind by the grappling iron of frequent repetition.

So long as superficial qualifications satisfy the medical student's ambition, he has not the adequate inducement to undergo the labor which is the purchase money of all acquirements that are valuable and solid. He cannot take the boon and shirk the condition. Fame is the reward of toil; and he is indeed deceived who expects to command resources without the trouble of acquiring them, or to rise to eminence upon a bed of roses, or to win, without an effort, that laurel-wreath which crowns none but those who earn it by their diligence.

> "Love, fame, esteem, 'tis labor must acquire;
> The smiling offspring of a rigid sire."

He, therefore, who would attain to eminence, must keep pace with the progress of medical science. As the age moves onward, he must move on with it, or be left behind. But whilst ignorance, in a period of such general intelligence, would indeed be a reproach to almost any man, let it also be remembered that unusual efforts are required of those who aspire to rise above mediocrity, or to shine with superior lustre in the medical firmament. They, and they only, can hope to be stars in an age like this, of whom it may be truly said, as regards their preparatoıy course,

> "See how the matchless youths their hours improve,
> And in the glorious way to knowledge move,
> Eager for fame, prevent the rising sun,
> And watch the midnight labor of the moon."

But while one poet thus stimulates your ambition to excel in learning as the only sure groundwork of power and distinction, another urges impressively the important truth that

> "Not in mental but in moral worth
> God excellence placed, and only to the good,
> The virtuous, grants happiness below."

However desirable, therefore, it may be to be distinguished, it is infinitely more important to be virtuous. The pillar whose base has no foundation, can give no support to the dome under which its head is placed. The pioneer's work must be done well if the steep and difficult paths to

> "Where Fame's proud temple shines afar"

are to be conquered.

While you are for a time dependent, to a great extent, upon us for your opinions, we desire you to reason closely upon what we teach. I can, however, assure you, gentlemen, that medical science is founded upon incontrovertible facts. Anatomy, the corner stone of medicine, is itself a science of facts; its principles are true to a demonstration.

For a long time our fathers groped in darkness. Medical men studied Anatomy by stealth. Indeed, among the ancients there was, by certain sects, a great deal of ridiculous wrangling as related to the utility of a knowledge of Anatomy. But some persevered and developed fact after fact, and finally Harvey discovered the circulation of the blood, and the wonderful uses of the heart, and traced the movements of the life-giving fluid carrying its nitrogen and oxygen to the exhausted tissues of all parts of the body. This gave a new and mighty impulse to the science, and settled many questions that had before been puzzling enigmas to medical men. And, to-day, Anatomy stands more complete in its details than any of the physical sciences, and, among scientific medical men, not a doubt of its value, as relates to medicine, is for a moment ever seriously discussed. But yet, as important and essential, as indispensable as the knowledge of Anatomy is, it is amazing how much it is neglected by a majority of students. I hope, young gentlemen, this will not be the case with you. Let it sink into your hearts and be thoroughly impressed upon your minds, that every man who loses a patient without a correct knowledge of the structure of the human frame—the form, size, situation and relative position of the different organs in a healthy state, and the mutations which are produced by disease, and which alter the color, shape and relations of the parts as well as their structure, is morally responsible for the life of his victim—for victim he is to the ignorance of the physician to whom he entrusted his life. But, young gentlemen, there is no reason why any of you should incur so fearful a responsibility, because you will have abundant opportunities here for the study and attainment of Anatomical knowledge, and nothing but patient perseverance and industry are necessary on your part in order to secure it; and, believe me, without a knowledge of this branch of medical science, your labors for medical attainment and distinction will be comparatively in vain.

Physiology is based on a knowledge of the structure of the human organization, and teaches the vital functions of that organization —a beautiful and sublime study which, it is true, is not reduced, in every particular, to anything like positive precision, but yet, in its leading principles, it is a settled and complete science, a thorough knowledge of which is absolutely necessary to enable the practitioner to triumph over disease. Rapid as has been the march of this department of medical science; brilliant as the conquests have been made by its cultivators, there are yet trophies in reserve for the persevering student. All the laurels have not yet been won. It is for you to investigate these principles, and for some of you, I trust, to discover and develop new truths. We are wiser than those who have passed away. Every generation adds to the stock of human knowledge; and however the case may be with individuals, yet with the species there is no doubt that man, generation by generation, grows wiser and wiser. In the discovery of every new truth there is increase of knowledge, and every fresh fact that is discovered concerning the mysteries of the universe, or of the economy of the human system, is a clue leading from the very chambers of knowledge, which the discoverer leaves behind him to guide his followers. It is never lost; it marks the spot up to which he had arrived, and his experience serves us instead of knowledge; for we may at pleasure take up the thread and commence where he ended, lead where it may.

Therefore, gentlemen, because knowledge is progressive; because you are young and fresh in the race upon this sea of life; because you have the experience of the great lights that are now shedding their concentrated radiance upon your path, we have reason to expect of you conquests still more brilliant than even those that have adorned the records of the past.

You may say that there are no unexplored fields—that industrious laborers have wrought in them all. But you need not labor in untried fields. The gold digger of to-day applies new machinery and new systems in his mining operations to gold fields long abandoned as exhausted and worthless, and gathers rich rewards for his labor. Strive, then, for yourselves. Where others have reaped fifty fold, you may reap an hundred fold.

It sometimes happens that a medical agent does not receive the attention it should from the profossion, when some one-idea man takes hold of it and developes new therapeutical applications. Learn, gentlemen, from all such. There are many plants, native to our forests, which are not appreciated as they should be. Your attention may be called to them by persons who know nothing of the principles of botany and materia medica. You may gain something from the most ignorant. The man who thinks the moon no bigger than a cheese, may suggest some idea from which the thinking man elaborates some important truth. Therefore, young gentlemen, never allow yourselves to become prejudiced against a medicine, or any remedial agent, because it is claimed or used by quacks. Rid yourselves from all false pride and pretensions; stand on the firm foundation of common sense; direct your time and efforts to the search after truth, wherever it may be found.

And now, gentlemen, ere I close my remarks, I would say that nothing conduces more to the happiness and peace of mind of the physician than the consciousness that he has done his duty well. When the eve of life approaches, and the setting sun beams its last ray over us, how pleasant it must be to feel that suffering humanity has been benefited at our hands. We should, therefore, during our days of activity, remember our duties and perform them well. Something more ennobling—something of greater value than the love of lucre should actuate us to pursue the professional path we have chosen. When the pallid sufferer lies groaning on his bed, the mere knowledge that by his suffering we profit, should not induce us to attempt his cure. Sympathy for his affliction should spring unbidden in our hearts, and the same feelings which would arise from the sufferings of a brother, should be engendered in us towards any fellow-creature.

